# Evidence for intragenic recombination and selective sweep in an effector gene of *Phytophthora infestans*

**DOI:** 10.1111/eva.12629

**Published:** 2018-05-09

**Authors:** Lina Yang, Hai‐Bing Ouyang, Zhi‐Guo Fang, Wen Zhu, E‐Jiao Wu, Gui‐Huo Luo, Li‐Ping Shang, Jiasui Zhan

**Affiliations:** ^1^ State Key Laboratory of Ecological Pest Control for Fujian and Taiwan Crops Fujian Agriculture and Forestry University Fuzhou China; ^2^ Fujian Key Lab of Plant Virology Institute of Plant Virology Fujian Agriculture and Forestry University Fuzhou China; ^3^ Xiangyang Academy of Agricultural Sciences Xiangyang China; ^4^ Key Lab for Biopesticide and Chemical Biology Ministry of Education Fujian Agriculture and Forestry University Fuzhou China

**Keywords:** climate change, compensatory mutation, effector genes, evolution, *Phytophthora infestans*, recombination, selective sweep, temperature dependent

## Abstract

Effectors, a group of small proteins secreted by pathogens, play a critical role in the antagonistic interaction between plant hosts and pathogens through their dual functions in regulating host immune systems and pathogen infection capability. In this study, evolution in effector genes was investigated through population genetic analysis of Avr3a sequences generated from 96 *Phytophthora infestans* isolates collected from six locations representing a range of thermal variation and cropping systems in China. We found high genetic variation in the Avr3a gene resulting from diverse mechanisms extending beyond point mutations, frameshift, and defeated start and stop codons to intragenic recombination. A total of 51 nucleotide haplotypes encoding 38 amino acid isoforms were detected in the 96 full sequences with nucleotide diversity in the pathogen populations ranging from 0.007 to 0.023 (mean = 0.017). Although haplotype and nucleotide diversity were high, the effector gene was dominated by only three haplotypes. Evidence for a selective sweep was provided by (i) the population genetic differentiation (*G*_ST_) of haplotypes being lower than the population differentiation (*F*_ST_ *)* of SSR marker loci; and (ii) negative values of Tajima's D and Fu's FS. Annual mean temperature in the collection sites was negatively correlated with the frequency of the virulent form (Avr3a^EM^), indicating Avr3a may be regulated by temperature. These results suggest that elevated air temperature due to global warming may hamper the development of pathogenicity traits in *P. infestans* and further study under confined thermal regimes may be required to confirm the hypothesis.

## 1 INTRODUCTION

Pathogens and their host plants are engaged in a never‐ending battle, in which pathogens are continuously evolving invasive mechanisms to break plant defense systems and host plants responding with the constant development of new protection to prevent or mitigate damage (Zhan, Thrall, & Burdon, 2014; Zhan, Thrall, Papaïx, Xie, & Burdon, 2015). Many plant pathogens interact with their hosts following the widely accepted gene‐for‐gene model. This interaction model hypothesizes that plant immune reactions are triggered by effectors, a group of small proteins secreted by pathogens during infection, when they are recognized by receptor proteins produced by corresponding host resistance genes (Hein, Gilroy, Armstrong, & Birch, 2009; Jones & Dangl, 2006). This theory suggests that effector genes may reduce pathogen fitness and therefore are selected against during pathogen evolution. However, recent molecular and functional analyses indicate that many effector proteins are essential components of infection and may manipulate plant cellular processes to increase host susceptibility and suppress host immune systems (Boevink et al., 2016; Wang et al., 2015; Yang, McLellan, et al., 2016). Consequently, effector genes are expected to evolve differently to other parts of pathogen genomes in reflecting this antagonistic coevolution between the pathogen and host and the trade‐off of the advantages and disadvantages any effector change may cause to the pathogen population. On the one hand, effector genes might be expected to evolve at an escalated rate in order to effectively and quickly escape host recognition systems and increase the invasive, survival, and reproductive chances of the pathogen. However, at the same time they are potentially constrained by the need to retain features critical to the biological and ecological adaptation of pathogens.

Effector genes commonly lie in gene‐sparse, transposon‐rich regions of the pathogen genome (Haas et al., 2009; Raffaele & Kamoun, 2012). This physical location provides effector genes a unique opportunity to generate sequence duplication. Functional redundancy encoded by multiple copies of effector genes relaxes selective pressure on one or more of the gene copies, which in turn allows more frequent and abrupt mutations to occur without severe impact on the fitness of the pathogen. Indeed, multiple mutation mechanisms including base substitution, deletion, pseudogenization, and transcriptional silencing have been documented in effector genes (Cooke et al., 2012; Raffaele & Kamoun, 2012). Interaction of these mutation events results in higher genetic variation in effector genes than in the rest of pathogen genome (Karasov, Horton, & Bergelson, 2014; Raffaele & Kamoun, 2012) and enhances response to selection driven by the change in host defense systems. Consequently, virulent types of effector genes can emerge quickly from avirulent types in the plant pathogen populations, leading to the breakdown of many major gene‐mediated plant resistances within a few years of their commercialization (Cooke et al., 2012; Pilet, Pellé, Ellisseche, & Andrivon, 2005).

In addition to their genetic characters, other biological and ecological processes may also influence genetic variation and the evolutionary trajectory of effector genes in nature. In a biological context, genetic variation of effector genes can be affected by recombination occurring either within a single gene (intragenic recombination) or between genes (intergenic recombination). Intragenic recombination can increase genetic variation of effector genes directly through generating new sequences or indirectly by reducing allelic loss associated with random genetic drift and hitchhiking selection. In an ecological context, selection driven by host defenses may be confounded by other biological and environmental factors such as temperature in determining the genetic variation and potential evolutionary landscapes of effector genes. Temperature can exert a critical influence on all aspects of chemical, biological, ecological, and evolutionary processes. It can affect survival, reproduction, and transmission of plants and pathogens both individually and interactively. Indeed, temperature‐mediated rate of spontaneous mutations has been documented in some species (Matsuba, Ostrow, Salomon, Tolani, & Baer, 2013). In host–pathogen interaction, temperature can regulate plant resistance responses to pathogens as well as the expression and competition of effector proteins (Banta, Bohne, Lovejoy, & Dostal, 1998).

*Phytophthora infestans* (Mont) de Bary is a pathogenic oomycete causing late blight diseases of potatoes and tomatoes. It distributes globally and can cost billions of dollars economic losses annually in the potato industry alone (Fry, 2008). In the potato–*P. infestans* interaction, resistance in the host is triggered by corresponding effector proteins released by the pathogen. Molecular genomic analyses confirm that *P. infestans* secretes a wide range of effector proteins into extracellular space (apoplastic effectors) or cell cytoplasm (cytoplasmic effectors). The pathogen genome contains at least 550 effectors all earmarked with a conserved N‐terminal motif of Arginine–X–Leucine–Arginine (RXLR), where X can be one of any 20 amino acids (Haas et al., 2009). However, this RXLR effector repertoire is likely expanding, contributing to the enormous size of the *P. infestans* genome (Haas et al., 2009; Raffaele & Kamoun, 2012). Biochemical and virulent functions of some RXLR effectors in *P. infestans* have been characterized recently (Bozkurt et al., 2011; King et al., 2014; McLellan et al., 2013; Wang et al., 2015; Yang, McLellan, et al., 2016).

Avr3a, recognized by the corresponding resistance gene R3a in the host plant, was the first effector gene characterized in *P. infestans* (Armstrong et al., 2005). It is a RXLR effector containing conserved W, Y, and L motifs in C‐terminal domains. These conserved motifs are believed to be generated by purifying selection imposed by the plant host over the course of the co‐evolutionary history of host and pathogen (Dou et al., 2008; Haas et al., 2009; Jiang & Tyler, 2008). The effector gene is also essential to pathogen infection. Silencing Avr3a compromised the pathogenicity of *P. infestans* in a susceptible potato cultivar and *Nicotiana benthamiana* (Bos et al., 2010; Vetukuri et al., 2011). Although both virulent and avirulent types of Avr3a existed in *P. infestans*, the former are more frequent, accounting for 60%–100% of Avr3a in natural populations (Armstrong et al., 2005; Cárdenas et al., 2011; Wu et al., 2016). Recently, several main groups of isoforms have been functional analyzed. Avr3a^EM^, containing amino acid residues S^19^, E^80^, M^103^, can evade recognition by plant hosts carrying R3a resistance gene (Bos et al., 2006) and is the most dominant virulent type. Avr3a^KI^, containing amino acid residues C^19^, K^80^, and I^103^, is an avirulent type. This isoform can be recognized by potato hosts with R3a resistance; it also promotes pathogen pathogenicity by suppressing the programmed cell death of resistant host plants. Avr3a^KI/147del^ and Avr3a^KI/Y147S^ all demonstrate the ability to activate the hypersensitive response in plants carrying R3a resistance but do not enhance the ability of the pathogen to infect (Bos, Chaparro‐Garcia, Quesada‐Ocampo, Gardener, & Kamoun, 2009).

Research on effector genes mushroomed in the last decade. The majority of these studies have focused on the molecular and functional dissection of effector genes. Population genetic analyses of effector genes and their interactions with environmental factors such as temperature are limited but urgently required to properly understand the evolutionary and epidemic behaviors of plant pathogens under a global warming scenario. In this study, we used Avr3a in *P. infestans* as a model to test the hypothesis that intragenic recombination and thermal conditions play an important role in the evolution of effector genes in plant pathogens. The specific objectives of this study were as follows: (i) to determine evolutionary mechanisms generating genetic variation of Avr3a; (ii) to infer the contribution of natural selection to the spatial population genetic dynamics of Avr3a; and (iii) to evaluate the potential impact of temperature on the spatial distribution and evolution of Avr3a.

## 2 MATERIALS AND METHODS

### 2.1 Pathogen isolates

Ninety‐six isolates (Table 1) each with a distinct genotype determined previously by molecular and phenotypic markers (Zhu et al., 2015; Yang, Zhu, et al., 2016) were included in this study. The isolates were collected from six commercial fields, one each from six regions located in Anshun (Guizhou), Fuzhou (Fujian), Guyuan (Ningxia), Kunming (Yunnan), Nanning (Guangxi), and Yangshuo (Ningxia) in the early stage of epidemics between 2010 and 2012. Gansu and Ningxia populations were kindly provided by Professor Weixing Shan at the Northwest A&F University. Detailed information on pathogen collection and isolation can be found in previous publications (Yang, Zhu, et al., 2016; Zhu et al., 2015). Briefly, infected leaf samples (one per plant) were collected randomly from potato plants at a spacing of at least 100 cm. Infected leaves were kept separate to prevent cross‐contamination and transferred to the laboratory on ice within 24 hr. In the laboratory, infected leaves were rinsed with sterilized water and single‐mycelium isolates secured from sporulating lesions using an inoculating needle. The isolates were purified by repeatedly transferring a single piece of mycelium to fresh medium three times. Only one single‐mycelium isolate was kept from each infected leaf.

**Table 1:** Geographic coordinates, annual mean temperatures, and summary of statistics for Avr3a in six *Phytophthora infestans* populations

Population	Location	AMT^a^	No. of sequences	*S* b	No. of haplotypes	Haplotype diversity	Nucleotide diversity
Ningxia	Guyuan	7.0	19	25	8	0.719	0.007
Gansu	Tianshui	11.7	17	46	11	0.912	0.014
Guizhou	Anshun	14.7	17	46	11	0.882	0.021
Yunnan	Kunming	15.6	17	55	13	0.926	0.018
Fujian	Fuzhou	20.5	11	45	10	0.982	0.019
Guangxi	Nanning	22.6	15	43	10	0.857	0.023
Total			96	117	51	0.884	0.017

a AMT = Annual mean temperature in collection sites.

b S = Number of variable sites.

### 2.2 DNA extraction and Avr3a sequencing

*Phytophthora infestans* isolates were retrieved from long‐term storage and cultured on rye B agars at 18°C in the dark. Mycelia (~100 mg) were harvested 15 days after inoculation, transferred into sterile, 2‐mL centrifuge tubes, and lyophilized with a vacuum freeze dryer (Alpha1‐2, Christ, Germany). The lyophilized mycelia were ground to powder with a mixer mill (MM400, Retsch, Germany). Total DNA was extracted using a Plant gDNA Miniprep Kit (GD 2611, Biomiga, China) according to the manufacturer's instructions. The genomic DNA was suspended in 200 μL of ultrapure water and stored at − 20°C until use.

Genomic DNA was amplified with primers (F: 5′‐CCATGCGTCTGGCAATTATGCT‐3′, R: 5′‐CTGAAAACTAATATCCAGTGA‐3′, Armstrong et al., 2005). These primers were originally designed for Pex147 (Armstrong et al., 2005) but were successfully used to amplify Avr3a in a previous study (Cárdenas et al., 2011). PCRs were carried out in a 25 μl reaction volume using Gene Cycler^™^ (Bio‐Rad, Shanghai). Each reaction contained 1× PCR buffer, 100 μM dNTPs, 1 unit of Taq polymerase (TaKaRa Ex Taq^®^), 0.32 μM of primers and 20 ng of template DNA. PCR amplification was started with an initial denaturation step of 94°C for 4 min, followed by 35 cycles of 94°C for 40 s, 58°C for 1 min, 72°C for 1.5 min, and ended by an extension cycle of 72°C for 5 min. PCR products were isolated by electrophoresis and purified for single direction sequencing according to the manufacturer's instructions (QIAquick^®^ Gel Extraction Kit), ligated into T5 zero cloning vector and transformed into *Trans*1‐T1 competent cells by heat‐shock at 42°C for 30 s (pEASY^®^‐T5 Zero Cloning Kit). Three colonies were picked from each transformation and incubated in liquid LB media at 37°C with shaking. One colony was randomly picked and sequenced by GenScript Biological Technology Co., Ltd. (GenScript, Nanjing, China) using an ABI3730 automated DNA sequencer (Applied Biosystems, USA).

### 2.3 Population genetic analyses and gene networks

Avr3a isoforms were deduced from nucleotide sequences, and multiple sequence alignments were performed using the MUSCLE algorithm (Edgar, 2004) implemented in MEGA5 (Tamura et al., 2011). Haplotypes were reconstructed by PHASRE algorithm implemented in DnaSP 5.10 (Librado & Rozas, 2009). The DnaSP 5.10 program was also used to estimate haplotype diversity, nucleotide diversity, and overall population differentiation (*G*_ST_) in Avr3a. Haplotype and nucleotide diversities were estimated for each of the six populations as well as the combined population by pooling the sequences for individual populations. SSR data of the isolates were taken from previous publications (Wu et al., 2016; Yang, Zhu, et al., 2016), and overall population differentiation in the SSR marker loci was estimated by fixation index (*F*_ST_) using POPGENE 1.32 (http://www.ualberta.ca/~fyeh/popgene_download.html). The standardized deviation of *K*_ST_ was generated by bootstrapping with 100 replicates and used to compare *G*_ST_ in the Avr3a gene and *F*_ST_ in SSR marker loci by a *t* test (Gao, Zou, Xie, & Zhan, 2017). A median‐joining (MJ) network illustrating genealogical relationships among haplotypes was generated using Network 5.0 (Bandelt, Forster, & Rohl, 1999).

### 2.4 Intragenic recombination, natural selection, and phylogenetic relationship

Putative intragenic recombination events and parental sequences were identified with seven algorithms (RDP, GENECONV, Bootscan, MaxChi, Chimaera, SiScan, and 3Seq) using the RDP4 suite (Martin, Murrell, Golden, Khoosal, & Muhire, 2015). The likelihood of putative recombination and parental sequence detections was corrected by a Bonferroni procedure with a cut‐off of *p *<* *.01, and only detections supported by at least four of the seven algorithms were retained. Recombination events were confirmed and displayed by similarity plots implemented in SimPlot 3.5.1 (Lole et al., 1999), a window size of 20 nucleotides and a step size of two nucleotides. The recombinant sequences were excluded from subsequent analysis of phylogenetic relationships.

Selective neutrality in Avr3a was evaluated by Tajima's *D* (Tajima 1989) and Fu's *F*_S_ (Fu, 1997) simultaneously using Arlequin 3.5 (Excoffier & Lischer, 2010). Recent selective sweep or purifying selection can cause an excess of rare polymorphisms to neutral expectation in sequences (Tajima 1989, Fu, 1997), leading to negative estimates of the two indexes in Avr3a. On the other hand, balancing or diversifying selection may generate positive estimates of the indexes as a result of deficiency of polymorphisms with low and high frequencies (Tajima 1989, Fu, 1997). The hypothesis of selective neutrality in Avr3a is retained if the estimated indexes did not differed significantly to the theoretical expectation of zeroes. Selective neutrality in Avr3a was also evaluated by the ratio of nonsynonymous (*d*N) to synonymous (*d*S) substitution (ω=*d*N/*d*S using four codon‐based algorithms implemented in HyPhy 2.10b (Pond et al. 2005) including FEL (fixed‐effects likelihood), IFEL (internal branches fixed‐effects likelihood), FUBAR (Fast Unconstrained Bayesian AppRoximation), and MEME (mixed effects model of evolution) (Yang 2007). Only sites simultaneously identified by FEL, IFEL, and MEME with *p* < .05 and >.95 posterior probability identified by FUBAR were considered to be under selection.

A Bayesian phylogenetic tree of Avr3a sequences was reconstructed using MrBayes 3.26 (Ronquist et al., 2012). The Bayesian analysis was carried out under the HKY substitution model, which was selected by MrModelTest (Nylander, 2008). Markov chain Monte Carlo chains (MCMC) were run for 10^6^ generations and sampled every 100 generations. Chain stationary and convergence of running parameters were checked using TRACER 1.6, and node support was evaluated with posterior probability generated from 10^5^ trees (burn‐in of 2500 trees).

### 2.5 Association between haplotype frequency and temperature

Thermal data for each collection site were downloaded from Weather Network (http://www.tianqi.com/). Annual mean temperature at collection sites was estimated using the air temperature across last 10 years as described previously (Yang, Zhu, et al., 2016). An association between isoform frequency (Avr3a^EM^) and the annual mean temperature at the collection sites was evaluated by Pearson's correlation (Lawrence & Lin, 1989).

## 3 RESULTS

### 3.1 Sequence variation in Avr3a

**Figure 1:**
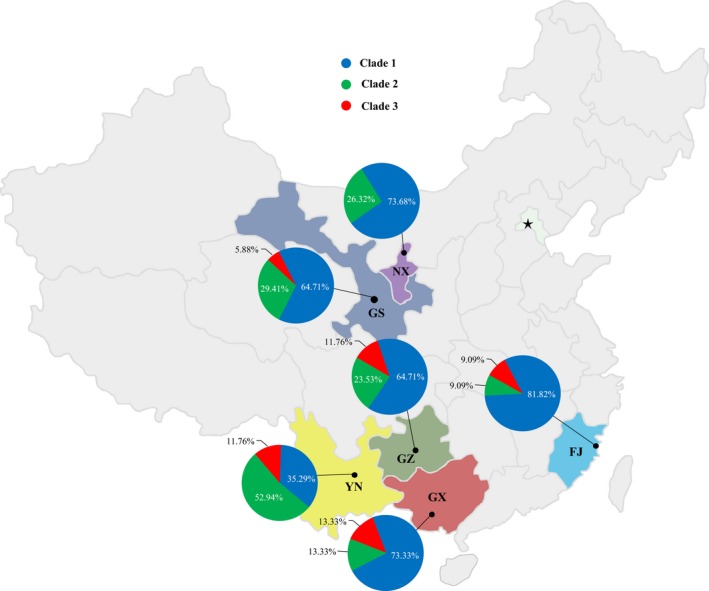
Frequency and spatial distribution of Avr3a haplotypes in the *Phytophthora infestans* isolates sampled from six geographical locations in China

**Figure 2:**
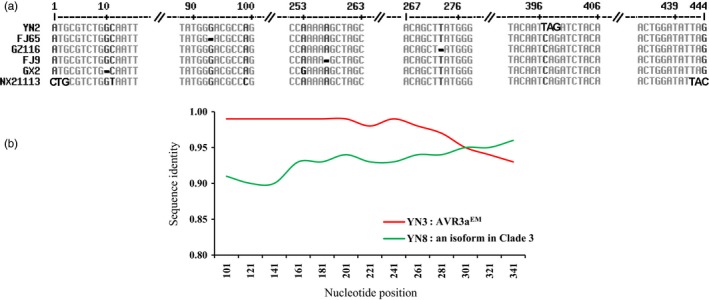
Mechanisms of generating genetic variation in Avr3a gene of *Phytophthora infestans*: (a) DNA sequences showing point mutations, early termination, single base deletion, and defeated start and stop codons in Avr3a. Point mutations in start and stop codons are highlighted with bold letters, and the single base deletions are highlighted with bold dash lines; (b) intragenic recombinant generated by YN3, an Avr3a^EM^ form, and YN8, an isoform in Clade 3

A total of 117 variable sites were detected in the 96 full nucleotide sequences, representing 11–19 from each of the six populations collected across China (Table 1, Figure 1). These variable sites formed 51 nucleotide haplotypes (Table 1) encoding 38 amino acid isoforms. The majority of sequence variations were generated by point mutations. Nearly 60% of the deduced amino acid isoforms differed only in one amino acid residue by a nonsynonymous mutation. Approximately 75% of the nonsynonymous mutations occurred in the effector domain and ~50% of those mutations were observed in the W and Y motifs. One isolate from Yunnan (YN2) had a mutation in the 397th nucleotide (C397T), generating an early termination stop codon causing a 12 C‐terminal amino acids truncation (Figure 2a). Four isolates from Fujian (FJ65 and FJ9), Guizhou (GZ116), and Guangxi (GZ116) populations had a single base deletion in the 10th, 93rd, 257th, and 273rd nucleotide, respectively (Figure 2a). Mutations in both start and stop codons were found in NX21113, a sequence from Ningxia (Figure 2a).

H‐1, H‐3, and H‐10 were the most common haplotypes, accounting for 16.7%, 30.2%, and 3.1% of the combined population, respectively (Figure 1). H‐1 and H‐3 were detected in all six populations, but H‐10 was only found in the pathogen populations from Southern China—in Fujian, Guangxi, Guizhou, and Yunnan (Figure 1). All other haplotypes were detected only once and were hence private to each population. The deduced amino acid isoform of H‐3 was identical to virulent Avr3a^EM^, while the amino acid sequence of H‐1 was one AA residue (R124G) different from virulent Avr3a^EM^. The deduced amino acid isoform of H‐10 was not found previously in the gene.

The haplotype diversity of nucleotide sequences in the six populations ranged from 0.719 to 0.982 with a grand mean of 0.884, when the 96 sequences from the six populations were combined. Nucleotide diversity of the populations ranged from 0.007 to 0.023 with a grand mean of 0.017 (Table 1). The Fujian population displayed the highest haplotype diversity, and the Guangxi population had the highest nucleotide diversity. The lowest haplotype and nucleotide diversities were found in the Ningxia population. The overall genetic differentiation across the six populations in haplotype (*G*_ST_) and eight SSR marker loci were 0.017 and 0.296, respectively. *G*_ST_ was significantly smaller than *F*_ST_ (*p* < .01) by a two‐tailed *t* test.

### 3.2 Haplotype network and geographical distribution

**Figure 3:**
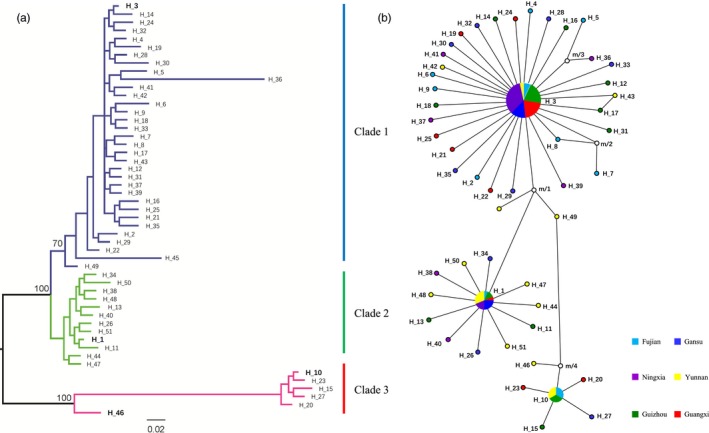
Phylogenetic relationship and haplotype network in the Avr3a sequences generated from 96 *Phytophthora infestans* isolates collected from six geographical locations in China; (a) phylogenetic relationships among the 51 Avr3a nucleotide haplotypes of *P. infestans* reconstructed by a Bayesian inference. Posterior probability of topology was generated by 1,000,000 bootstrapping and is shown on the three main clusters. The most common (H‐1, H‐3, and H‐10) and the recombinant (H46) sequences were highlighted with bold letters; (b) haplotype network of 51 Avr3a nucleotide sequences and circle sizes of the circles represent haplotype frequencies in populations

**Figure 4:**
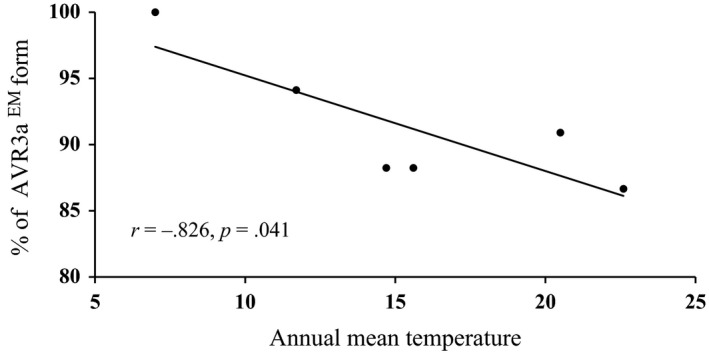
Correlation between annual mean temperature in collection sites and the frequency of Avr3a^EM^

The 51 haplotypes were grouped into three clades by Bayesian inference (Figure 3a). Clade 1 was composed of all haplotypes translated to the Avr3a^EM^ form, and Clade 2 was composed of haplotypes translated to isoforms similar to Avr3a^EM^ with a single AA residue change. Clade 3 consisted of four haplotypes, all except one (H‐46) translated to the same isoform. Nucleotide sequences from Gansu, Guizhou, Guangxi, and Fujian populations were found in all three clades with more than half being grouped into Clade 1. Sequences from the Yunnan population were also found in all three clades, but more than half were grouped in Clade 2. Sequences from the Ningxia population were only found in Clade 1 and Clade 2 (Figure 3). Nucleotide haplotypes within a clade were connected to each other by less than two base substitutions, and haplotypes among the clades were connected to each other by two to four steps of base substitution through a common progenitor sequence (m/1), which was not detected in the samples (Figure 1b). The combined frequency of Avr3a^EM^ (Clade 1) and its AA residue change alike (Clade 2) in the six populations ranged from 86.7% to 100% with an average of 91.4% (Figure 3b), while that of Clade 3 ranged from 0.0% to 13.3% with an average of 8.6% (Figure 3b). The frequency of the Avr3a^EM^ form was negatively correlated with the annual mean temperature of the collection sites (*r* = −.826, DF = 4, *p* = .041, Figure 4).

### 3.3 Intragenic recombination and neutrality test in Avr3a

Intragenic recombination was detected in one (YN17) of the sequences from the Yunnan population by four of the seven models with high confidence (GENECONV, *p* = 1.5^−4^; MAXCHI, *p* = 7.22^−9^; CHIMAERA, *p* = 5.66^−9^; and 3SEQ, *p* = 1.08^−13^). The recombinant was probably generated through interaction between YN3, an Avr3a^EM^ form sequence, and YN8, a new isoform in Clade 3 (Figure 2b). As with the recombinant sequence, both parental ones were detected in the Yunnan population. Tajima's *D* and Fu's *F*_S_ tests in the combined population from the six locations were −2.214 (*p* = .003) and −24.967 (*p* = .000), respectively. Both of these values were significantly lower than that expected under selective neutrality (zero), suggesting the existence of purifying selection acting on Avr3a. The ratio of nonsynonymous to synonymous substitution (dN/dS) was less than one in all variable sites but significance was found only in the 61st (*p* = .04) and 126th (*p* = .03) codons.

## 4 DISCUSSION

Genetic diversity of Avr3a gene was found to be high in the current study, and nucleotide diversity (Table 1) was positively correlated (*r* = .72, *p* = .10, data not shown) to SSR diversity (Qin et al., 2016). More than 50 nucleotide haplotypes were detected in 96 sequences, and the combined nucleotide diversity was 0.017 (Table 1). Effector genes are critical for the invasion, colonization, and reproduction of pathogens on hosts, and the finding of high genetic diversity in the effector gene Avr3a is consistent with the evolutionary hypothesis postulating that genes involving in antagonistic host‐pathogen coevolution have higher evolutionary rates compared to other genes as documented both in *P. infestans* (Cárdenas et al., 2011; de Vries et al., 2017) and many other species (Allen et al., 2004, 2008; Raffaele, Win, Cano, & Kamoun, 2010). This high evolutionary rate may well contribute to the rapid “breakdown” of many host resistances mediated by major genes (Cooke et al., 2012; Pilet et al., 2005). In previous studies, only two haplotypes formed by three SNPs were detected in 55 isolates of *P. infestans* (Armstrong et al., 2005) and six haplotypes generated by 12 SNPs were found in a different set of 88 sequences (Cárdenas et al., 2011). High haplotype diversity in the current study is unlikely due to artifacts generated by PCR amplification. The error rate for the standard Tag PCR‐based sequencing is 2.0 × 10^−5^ (Lundberg et al., 1991), and the error rate for the Ex Tag PCR‐based sequencing is approximately 4.5 times lower than that generated by standard Tag PCR based (http://www.clontech.com/MR/Products/PCR/High_Yield_PCR/Ex_Taq_DNA_Polymerase). Avr3a is 1,448 bp in length, and 96 haplotypes were sequenced in the study. Using this information, we estimated that the potential number of artifact sequences in our study was less than one (2 × 10^−5^ × 1,448 × 96/4.5 = 0.62). Furthermore, when isolates with “minor” haplotypes were resequenced with five independent clones, all but one “minor” haplotypes were recaptured in the new amplification.

Two factors may contribute to the differences in diversity found between the current and previous studies. Genetic variation, and therefore evolutionary potential, of populations can be underestimated when samples are over‐represented by identical genotypes (clones) generated by asexual reproduction of parents. This sample error is usually corrected using only one representative of the same genotype in the estimation of genetic variation (Zhan, Pettway, & McDonald, 2003). Unlike previous studies, isolates in the current study were prescreened molecularly and morphologically and only isolates with distinct genotypes were selected for sequence analysis (Wu et al., 2016). China also plants ~30% of the global potato acreage (http://faostat.fao.org/), thereby possibly hosting the largest *P. infestans* population in the world. Moreover, epidemiological analysis also suggests that the overall Chinese population of *P. infestans* has continued to expand in recent decades (unpublished data). Large population size coupled with its continuing expansion creates optimal conditions for the generation and maintenance of genetic variation in the Chinese pathogen population. Interestingly, no shared AA sequences were found between the current and previous studies except the Avr3a^EM^ virulent form (Armstrong et al., 2005; Cárdenas et al., 2011). In 2005, the frequency of Avr3a^KI^ accounted for ~15% of the population (55 isolates, Armstrong et al., 2005) but its frequency reduced to ~7% in 2011 (88 isolates, Cárdenas et al., 2011). In this study, no Avr3a^KI^ sequences were detected in 96 isolates, suggesting either this isoform has gradually disappeared from the pathogen populations or is patchily distributed in space. Instead, Avr3a^KI^ was replaced by a new isoforms deduced from H‐10, H‐15, H‐20, H‐23, and H‐27 with a sequence identical to Pex‐147‐2 (Armstrong et al., 2005).

Sequence analysis indicates that, as for many other effector genes (Cooke et al., 2012; Raffaele & Kamoun, 2012), various types of mutations are responsible for the level of genetic variation detected in Avr3a including point mutation, early termination, frameshift, and defeated start and stop codons (Figure 2a). Although nucleotide substitution was the main mechanism, other types of mutations account for ~15% of haplotype variation observed. Avr3a proteins secreted from *P. infestans* during infection trigger defense responses in hosts carrying corresponding resistance genes (Lo Presti et al., 2015) and are also important for the pathogenicity of plant pathogens by increasing host susceptibility or suppressing host immunity (Bos et al., 2010; Haas et al., 2009; Vetukuri et al., 2011). Although increasing the invasive opportunity of *P. infestans* due to the prevention of its recognition by potato hosts carrying with R3a gene, high mutation rates in Avr3a particular through pseudogenization also abruptly disrupt the normal biochemical functions of the gene. This result is consistent with previous hypothesis that gene duplication may occur frequently in Avr3a of *P. infestans* (Armstrong et al., 2005). Alternatively, it indicates secondary or tertiary mutations may occur in the genome to compensate for the fitness penalty association with loss of the Avr3a functions required by the pathogen. These compensatory mutations have been widely documented (Jochumsen et al., 2016; Sato et al., 2016). Continuing genome expansion (Raffaele & Kamoun, 2012) may provide a unique environment for the development of compensatory mutations leading to functional redundancy or epitasis (Birch et al., 2008; Oh et al., 2009) in the effector genes of *P. infestans*.

In addition to mutation, our results confirm that intragenic recombination also contributes to the generation of high genetic variation in Avr3a. Unlike intergenic recombination that generates new genotypes through random reshuffling of unaltered existing genes, intragenic recombination occurs among varying nucleotide sequences within a single‐gene locus, generating new allelic variation with potentially novel properties (Watt, 1972). When one considers that chromosomes are essentially simple strings of nucleotides separated into many genes of varying biological and ecological functions, recombination can potentially occur at any point regardless of whether it is within a particular gene or among genes. Hence, it is logical to expect that the number of intragenic recombination events may be similar to the number of intergenic ones although an unknown proportion of intragenic recombination may be deleterious or lethal. Despite this possibility, its role in the evolution of species, largely underestimated until recent advances in molecular and computation technologies, has been shown to be of great importance occurring in almost all species and kingdoms ranging from virus (He & Ding, 2012; He et al., 2010; Phan, Okitsu, Maneekarn, & Ushijima, 2007) to protists (Ferreira & Briones, 2012), fungi (Stergiopoulos et al., 2014), plants (Kelly et al., 2010; Ortega, Bošković, Sargent, & Tobutt, 2006; Städler & Delph, 2002), and animals (Godinho, Mendonça, Crespo, & Ferrand, 2006; Marthaler et al., 2014). In the current study, we have two lines of evidence to support the hypothesis that intragenic recombination also contributes to the high genetic variation and evolutionary potential of Avr3a. A chimeric sequence and its putative parents were detected in the isolates from the same population. This was supported by several algorithms with a high level of confidence (Figure 2b). In addition, a reticulation pattern in the haplotype network of some sequences (Figure 3b) also points to the possibility of intragenic recombination in Avr3a. Sequences chimeras could also be generated by joining two biological sequences together during PCR amplification, and this type of sequence chimeras usually disappear after one repeat of PCR re‐amplification (Smyth et al., 2010). However, the chimeric feature of YN17 sequence persists when the isolate was reamplified and resequenced, suggesting it is unlikely to be the artifact of PCR amplification.

Although large numbers of nucleotide haplotypes (51) and amino acid isoforms (38) were detected in the 96 sequences, only a single amino acid form (AVR3a^EM^) representing two nucleotide haplotypes (H‐1 and H‐3) dominated the *P. infestans* populations. All other isoforms including the new isoforms deduced from H‐10, H‐15, H‐20, H‐23, and H‐27 had a frequency of less than 5%. These results suggest that Avr3a gene might have experienced a selective sweep during its evolutionary history (Armstrong et al., 2005). Selective sweeps, usually associated with purifying selection, occur when environments select for the same traits/genotypes among populations inhabiting different ecological niches, thereby reducing genetic differentiation in related traits/genotypes among geographic populations (Zhan et al. 2004; Karasov et al., 2014). The hypothesis of a selective sweep occurring in the effector gene was supported by a comparative population genetic analysis of Avr3a and SSR marker loci which showed that genetic differentiation in Avr3a (assessed through the *G*_ST_ statistic) was significantly lower than genetic differentiation in the SSR marker loci (assessed through the *F*_ST_ statistic, Zhan et al., 2005). Furthermore, neutrality tests showed both Tajima's D and Fu's FS to be significantly less than zero. Lower genetic differentiation in Avr3a than the SSR marker loci was also detected previously by phenotypic (virulence) analysis of *P. infestans* isolates on potato differentials (Wu et al., 2016). It is likely that H‐1 (Avr3a^EM^) survived the selective sweep and other haplotypes (isoforms) were derived from the progenitor through mutation and intragenic recombination. The observation of selective sweep indicates Avr3a is an important gene contributing to survival, reproduction, and transmission of *P. infestans*, consisting with functional analysis of the gene (Bos et al., 2010). Indeed, six isolates with nonfunctional Avr3a sequences (Figure 2a) produced ~60% less disease on a susceptible cultivar (data not shown).

Host resistance is expected to be the primary force responsible for the observed pattern of natural selection in Avr3a. It is likely that potato cultivars with R3a gene have been intentionally or unintentionally used across many regions of the world, selecting for Avr3a isoforms able to circumvent the resistance. Indeed, the resistance gene has been detected in many potato cultivars recently (Nowicki, Foolad, Nowakowska, & Kozik, 2012; Zhu, Li, Vossen, Visser, & Jacobsen, 2012). In addition, environmental factors such as temperature may interact with host resistance influencing the fitness of Avr3a in *P. infestans*. Temperature is one of the most important environmental parameters crucially impacting many aspects of host–pathogen interactions including host susceptibility (Menna, Nguyen, Guttman, & Desveaux, 2015), pathogen density (Mikkelsen, Jørgensen, & Lyngkjær, 2015), and effector gene expression (Banta et al., 1998). The hypothesis of temperature‐mediated Avr3a fitness is supported by significant associations of isoform frequency with the mean annual temperature in the sample sites (Figure 4). Like other studies (Stefansson, Willi, Croll, & McDonald, 2014; Yang, Zhu, et al., 2016), we correlated Avr3a frequency to annual mean temperature rather than mean temperature in growing season for two reasons: (i) Pathogen adaptation to temperature occurs both in the epidemic and saprotrophic phase; and (ii) the length of the growing season is difficult to determine for many pathogens with multiple hosts due to cross‐species transmission. Regardless, a similar pattern of association was found when the mean temperature of potato growing season was used but the level of significance was reduced (data not shown). This result suggests that the pathogen with the Avr3a^EM^ form may adapt better to lower than higher temperatures. Avr3a^EM^ cannot be recognized by R3a (Armstrong et al., 2005) and therefore is a virulent type of Avr3a. Indeed, most isolates with Avr3a^EM^ sequences induced late blight symptoms on R3a differential plants in an assay (Wu et al., 2016). For example, Avr3a^EM^ (Clade 1 and 2) in isolates from Ningxia and Gansu accounted 100% and 94% of the populations, respectively, which correlated well with the frequency of virulent phenotypes. Isolates from Fujian matched least between genetic and phenotypic data. In this population, Avr3a^EM^ and virulent phenotype accounted for ~90% and ~70%, respectively. The lower phenotypic than genotypic frequency in Fujian and other three populations likely results from recessive nature of Avr3a^EM^ or gain of function mutants (Armstrong et al., 2005; Bos et al., 2009). This result indicates that increases in air temperature during global warming may slow the appearance of Avr3a and other virulence factors in *P. infestans* (Wu et al., 2016).

Directional selection on plant pathogens, which can generate selective sweep under extreme conditions, is the main mechanism driving the rapid breakdown of host resistance observed in agriculture. Unlike intragenic recombination, the direction and magnitude of selection on plant pathogens can be manipulated through the change in agricultural practices. To slow down the evolution of plant pathogens and achieve durable resistance in hosts in modern agriculture, it is important to create field conditions favoring disruptive selection through the evolutionary deployment of host resistance such as a cultivar mixture (Zhan et al., 2014, 2015). In addition, elevated air temperature associated with anthropogenic activities may greatly threaten food security due to extended seasonality supporting more plant diseases or enhanced pathogen growth thereby driving more intense disease epidemics. The negative association between virulence frequency of plant pathogens and local temperature found in the current and previous studies (Wu et al., 2016) raises the possibility that increasing air temperature during global warming may negatively affect the generation and maintenance of novel virulence genes in *P. infestans*, extending the life span of resistance genes. However, further investigations by functional, competition, and experimental evolution analyses of effector genes under controlled temperatures are required to confirm the result.

## CONFLICT OF INTEREST

None declared.

## DATA ARCHIVING STATEMENT

Haplotypes generated in this study are deposited in GenBank under accession numbers: MH043150 (FJ‐F002, hap‐1), MH043151 (FJ‐F041, hap‐3), MH043152 (FJ‐F050, hap‐4), MH043153 (FJ‐F052, hap‐5), MH043154 (FJ‐F057, hap‐6), MH043155 (FJ‐F064, hap‐7), MH043156 (FJ‐F103, hap‐9), MH043157 (FJ‐F105, hap‐10), MH043158 (GZ‐GA005, hap‐11), MH043159 (GZ‐GA007, hap‐12), MH043160 (GZ‐GA009, hap‐13), MH043161 (GZ‐GA014, hap‐14), MH043162 (GZ‐GA029, hap‐15), MH043163 (GZ‐GA038, 16), MH043164 (GZ‐GA041, hap‐17), MH043165 (GN‐GN003, hap‐20), MH043166 (GN‐GN008, hap‐21), MH043167 (GN‐GN010, hap‐22), MH043168 (GN‐GN013, hap‐23), MH043169 (GN‐GN016, 24), MH043170 (GN‐GN033, hap‐25), MH043171 (GN‐GN057, hap‐26), MH043172 (GS‐Pd11201, hap‐27), MH043173 (GS‐Pd11220, hap‐28), MH043174 (GS‐Pd11239, hap‐29), MH043175 (GS‐Pd11248, hap‐30), MH043176 (GS‐Pd11251, hap‐31), MH043177 (GS‐Pd11322, hap‐32), MH043178 GS (Pd11330, hap‐33), MH043179 (GS‐Pd13213, hap‐34), MH043180 (GS‐Pd13220, hap‐35), MH043181 (NX‐Pd21203, hap‐37), MH043182 (NX‐Pd213100, hap‐38), MH043183 (NX‐Pd213183, hap‐39), MH043184 (NX‐Pd21366, hap‐40), MH043185 (NX‐Pd21418, hap‐41), MH043186 (YN‐YN003, hap‐43), MH043187 (YN‐YN009, hap‐44), MH043188 (YN‐YN015, hap‐45), MH043189 (YN‐YN017, hap‐46), MH043190 (YN‐YN021, hap‐47), MH043191 (YN‐YN038, hap‐48), MH043192 (YN‐YN055, hap‐49), MH043193 (YN‐YN068, hap‐50), MH043194 (YN‐YN071, hap‐51).

## References

[eva12629-bib-0001] Allen, R. L. , Bittner‐Eddy, P. D. , Grenville‐Briggs, L. J. , Meitz, J. C. , Rehmany, A. P. , Rose, L. E. , & Beynon, J. L. (2004). Host‐parasite coevolutionary conflict between Arabidopsis and downy mildew. Science, 306, 1957–1960. 10.1126/science.1104022 15591208

[eva12629-bib-0002] Allen, R. L. , Meitz, J. C. , Baumber, R. E. , Hall, S. A. , Lee, S. C. , Rose, L. E. , & Beynon, J. L. (2008). Natural variation reveals key amino acids in a downy mildew effector that alters recognition specificity by an arabidopsis resistance gene. Molecular Plant Pathology, 9, 511–523. 10.1111/j.1364-3703.2008.00481.x 18705864 PMC6640421

[eva12629-bib-0003] Armstrong, M. R. , Whisson, S. C. , Pritchard, L. , Bos, J. I. , Venter, E. , Avrova, A. O. , … Hamlin, N. (2005). An ancestral oomycete locus contains late blight avirulence gene Avr3a, encoding a protein that is recognized in the host cytoplasm. Proceedings of the National Academy of Sciences of the United States of America, 102, 7766–7771. 10.1073/pnas.0500113102 15894622 PMC1140420

[eva12629-bib-0004] Bandelt, H. J. , Forster, P. , & Rohl, A. (1999). Median‐joining networks for inferring intraspecific phylogenies. Molecular Biology and Evolution, 16, 37–48. 10.1093/oxfordjournals.molbev.a026036 10331250

[eva12629-bib-0005] Banta, L. M. , Bohne, J. , Lovejoy, S. D. , & Dostal, K. (1998). Stability of the *Agrobacterium tumefaciens* VirB10 protein is modulated by growth temperature and periplasmic osmoadaption. Journal of Bacteriology, 180, 6597–6606. 9852004 10.1128/jb.180.24.6597-6606.1998 PMC107763

[eva12629-bib-0006] Birch, P. R. , Boevink, P. C. , Gilroy, E. M. , Hein, I. , Pritchard, L. , & Whisson, S. C. (2008). Oomycete RXLR effectors: Delivery, functional redundancy and durable disease resistance. Current Opinion in Plant Biology, 11, 373–379. 10.1016/j.pbi.2008.04.005 18511334

[eva12629-bib-0007] Boevink, P. C. , Wang, X. , McLellan, H. , He, Q. , Naqvi, S. , Armstrong, M. R. , … Birch, P. R. (2016). A *Phytophthora infestans* RXLR effector targets plant PP1c isoforms that promote late blight disease. Nature Communications, 7, 10311 10.1038/ncomms10311 PMC4740116 26822079

[eva12629-bib-0008] Bos, J. I. , Armstrong, M. R. , Gilroy, E. M. , Boevink, P. C. , Hein, I. , Taylor, R. M. , … Dixelius, C. (2010). *Phytophthora infestans* effector AVR3a is essential for virulence and manipulates plant immunity by stabilizing host E3 ligase CMPG1. Proceedings of the National Academy of Sciences of the United States of America, 107, 9909–9914. 10.1073/pnas.0914408107 20457921 PMC2906857

[eva12629-bib-0009] Bos, J. I. , Chaparro‐Garcia, A. , Quesada‐Ocampo, L. M. , Gardener, B. B. M. , & Kamoun, S. (2009). Distinct amino acids of the *Phytophthora infestans* effector AVR3a condition activation of R3a hypersensitivity and suppression of cell death. Molecular Plant‐Microbe Interactions, 22, 269–281. 10.1094/MPMI-22-3-0269 19245321

[eva12629-bib-0010] Bos, J. I. , Kanneganti, T. D. , Young, C. , Cakir, C. , Huitema, E. , Win, J. , … Kamoun, S. (2006). The C‐terminal half of *Phytophthora infestans* RXLR effector AVR3a is sufficient to trigger R3a‐mediated hypersensitivity and suppress INF1‐induced cell death in Nicotiana benthamiana. The Plant Journal, 48, 165–176. 10.1111/j.1365-313X.2006.02866.x 16965554

[eva12629-bib-0011] Bozkurt, T. O. , Schornack, S. , Win, J. , Shindo, T. , Ilyas, M. , Oliva, R. , … Kamoun, S. (2011). *Phytophthora infestans* effector AVRblb2 prevents secretion of a plant immune protease at the haustorial interface. Proceedings of the National Academy of Sciences of the United States of America, 108, 20832–20837. 10.1073/pnas.1112708109 22143776 PMC3251060

[eva12629-bib-0012] Cárdenas, M. , Grajales, A. , Sierra, R. , Rojas, A. , González‐Almario, A. , Vargas, A. , … Bernal, A. (2011). Genetic diversity of *Phytophthora infestans* in the Northern Andean region. BMC Genetics, 12, 1. 21303555 10.1186/1471-2156-12-23 PMC3046917

[eva12629-bib-0013] Cooke, D. E. , Cano, L. M. , Raffaele, S. , Bain, R. A. , Cooke, L. R. , Etherington, G. J. , … Grünwald, N. J. (2012). Genome analyses of an aggressive and invasive lineage of the Irish potato famine pathogen. PLoS Pathogens, 8, e1002940 10.1371/journal.ppat.1002940 23055926 PMC3464212

[eva12629-bib-0014] Dou, D. , Kale, S. D. , Wang, X. , Chen, Y. , Wang, Q. , Wang, X. , … McDowell, J. M. (2008). Conserved C‐terminal motifs required for avirulence and suppression of cell death by *Phytophthora sojae* effector Avr1b. The Plant Cell, 20, 1118–1133. 10.1105/tpc.107.057067 18390593 PMC2390733

[eva12629-bib-0016] Edgar, R. C. (2004). MUSCLE: Multiple sequence alignment with high accuracy and high throughput. Nucleic Acids Research, 32, 1792–1797. 10.1093/nar/gkh340 15034147 PMC390337

[eva12629-bib-0017] Excoffier, L. , & Lischer, H. E. (2010). Arlequin suite Version 3.5: A new series of programs to perform population genetics analyses under Linux and Windows. Molecular Ecology Resources, 10, 564–567. 10.1111/j.1755-0998.2010.02847.x 21565059

[eva12629-bib-0018] Ferreira, R. C. , & Briones, M. R. S. (2012). Phylogenetic evidence based on *Trypanosoma cruzi* nuclear gene sequences and information entropy suggest that inter‐strain intragenic recombination is a basic mechanism underlying the allele diversity of hybrid strains. Infection, Genetics and Evolution, 12, 1064–1071. 10.1016/j.meegid.2012.03.010 22449773

[eva12629-bib-0019] Fry, W. (2008). *Phytophthora infestans*: The plant (and R gene) destroyer. Molecular Plant Pathology, 9, 385–402. 10.1111/j.1364-3703.2007.00465.x 18705878 PMC6640234

[eva12629-bib-0020] Fu, Y. X. (1997). Statistical tests of neutrality of mutations against population growth, hitchhiking and background selection. Genetics, 147, 915–925. 9335623 10.1093/genetics/147.2.915 PMC1208208

[eva12629-bib-0021] Gao, F. L. , Zou, W. C. , Xie, L. H. , & Zhan, J. S. (2017). Adaptive evolution and demographic history contribute to the divergent population genetic structure of Potato virus Y between China and Japan. Evolutionary Applications, 10, 10.1111/eva.12459 PMC5367074 28352297

[eva12629-bib-0022] Godinho, R. , Mendonça, B. , Crespo, E. G. , & Ferrand, N. (2006). Genealogy of the nuclear β‐fibrinogen locus in a highly structured lizard species: Comparison with mtDNA and evidence for intragenic recombination in the hybrid zone. Heredity, 96, 454–463. 10.1038/sj.hdy.6800823 16598190

[eva12629-bib-0023] Haas, B. J. , Kamoun, S. , Zody, M. C. , Jiang, R. H. , Handsaker, R. E. , Cano, L. M. , … Bozkurt, T. O. (2009). Genome sequence and analysis of the Irish potato famine pathogen *Phytophthora infestans* . Nature, 461, 393–398. 10.1038/nature08358 19741609

[eva12629-bib-0024] He, C. Q. , & Ding, N. Z. (2012). Discovery of severe fever with thrombocytopenia syndrome bunyavirus strains originating from intragenic recombination. Journal of Virology, 86, 12426–12430. 10.1128/JVI.01317-12 22933273 PMC3486477

[eva12629-bib-0025] He, C. Q. , Ding, N. Z. , He, M. , Li, S. N. , Wang, X. M. , He, H. B. , … Guo, H. S. (2010). Intragenic recombination as a mechanism of genetic diversity in bluetongue virus. Journal of Virology, 84, 11487–11495. 10.1128/JVI.00889-10 20702614 PMC2953192

[eva12629-bib-0026] Hein, I. , Gilroy, E. M. , Armstrong, M. R. , & Birch, P. R. J. (2009). The zig‐zag‐zig in oomycete–plant interactions. Molecular Plant Pathology, 10, 547–562. 10.1111/j.1364-3703.2009.00547.x 19523107 PMC6640229

[eva12629-bib-0027] Jiang, R. H. Y. , & Tyler, B. M. (2008). RXLR effector reservoir in two *Phytophthora* species is dominated by a single rapidly evolving superfamily with more than 700 members. Proceedings of the National Academy of Sciences of the United States of America, 105, 4874–4879. 10.1073/pnas.0709303105 18344324 PMC2290801

[eva12629-bib-0028] Jochumsen, N. , Marvig, R. L. , Damkiær, S. , Jensen, R. L. , Paulander, W. , Molin, S. , … Folkesson, A. (2016). The evolution of antimicrobial peptide resistance in *Pseudomonas aeruginosa* is shaped by strong epistatic interactions. Nature Communications, 7, 13002 10.1038/ncomms13002 PMC5494192 27694971

[eva12629-bib-0029] Jones, J. D. G. , & Dangl, J. L. (2006). The plant immune system. Nature, 444, 323–329. 10.1038/nature05286 17108957

[eva12629-bib-0030] Karasov, T. L. , Horton, M. W. , & Bergelson, J. (2014). Genomic variability as a driver of plant–pathogen coevolution? Current Opinion in Plant Biology, 18, 24–30. 10.1016/j.pbi.2013.12.003 24491596 PMC4696489

[eva12629-bib-0031] Kelly, L. J. , Leitch, A. R. , Clarkson, J. J. , Hunter, R. B. , Knapp, S. , & Chase, M. W. (2010). Intragenic recombination events and evidence for hybrid speciation in Nicotiana (Solanaceae). Molecular Biology and Evolution, 27, 781–799. 10.1093/molbev/msp267 19897524

[eva12629-bib-0032] King, S. R. , McLellan, H. , Boevink, P. C. , Armstrong, M. R. , Bukharova, T. , Sukarta, O. , … Banfield, M. J. (2014). *Phytophthora infestans* RXLR effector PexRD2 interacts with host MAPKKKε to suppress plant immune signalling. Plant Cell, 26, 1345–1359. 10.1105/tpc.113.120055 24632534 PMC4001388

[eva12629-bib-0033] Lawrence, I. , & Lin, K. A. (1989). A concordance correlation coefficient to evaluate reproducibility. Biometrics, 45, 255–268. 2720055

[eva12629-bib-0034] Librado, P. , & Rozas, J. (2009). DnaSP v5: A software for comprehensive analysis of DNA polymorphism data. Bioinformatics, 25, 1451–1452. 10.1093/bioinformatics/btp187 19346325

[eva12629-bib-0035] Lo Presti, L. , Lanver, D. , Schweizer, G. , Tanaka, S. , Liang, L. , Tollot, M. , … Kahmann, R. (2015). Fungal effectors and plant susceptibility. Annual Review of Plant Biology, 66, 513–545. 10.1146/annurev-arplant-043014-114623 25923844

[eva12629-bib-0036] Lole, K. S. , Bollinger, R. C. , Paranjape, R. S. , Gadkari, D. , Kulkarni, S. S. , Novak, N. G. , … Ray, S. C. (1999). Full‐length human immunodeficiency virus type 1 genomes from subtype C‐infected seroconverters in India, with evidence of intersubtype recombination. Journal of Virology, 73, 152–160. 9847317 10.1128/jvi.73.1.152-160.1999 PMC103818

[eva12629-bib-0037] Lundberg, K. S. , Shoemaker, D. D. , Adamsb, M. W. W. , Short, J. M. , Serge, J. A. , & Mathur, E. J. (1991). High‐fidelity amplification using a thermostable DNA polymerase isolated from *Pyrococcus furiosus* . Gene, 108, 1–6. 10.1016/0378-1119(91)90480-Y 1761218

[eva12629-bib-0038] Marthaler, D. , Suzuki, T. , Rossow, K. , Culhane, M. , Collins, J. , Goyal, S. , … Matthijnssens, J. (2014). VP6 genetic diversity, reassortment, intragenic recombination and classification of rotavirus B in American and Japanese pigs. Veterinary Microbiology, 172, 359–366. 10.1016/j.vetmic.2014.05.015 24970362

[eva12629-bib-0039] Martin, D. P. , Murrell, B. , Golden, M. , Khoosal, A. , & Muhire, B. (2015). DP4: Detection and analysis of recombination patterns in virus genomes. Virus Evolution, 1, vev003. 27774277 10.1093/ve/vev003 PMC5014473

[eva12629-bib-0040] Matsuba, C. , Ostrow, D. G. , Salomon, M. P. , Tolani, A. , & Baer, C. F. (2013). Temperature, stress and spontaneous mutation in *Caenorhabditis briggsae* and *Caenorhabditis elegans* . Biology Letters, 23, 20120334. 10.1098/rsbl.2012.0334 PMC3565477 22875817

[eva12629-bib-0041] McLellan, H. , Boevink, P. C. , Armstrong, M. R. , Pritchard, L. , Gomez, S. , Morales, J. , … Birch, P. R. (2013). An RxLR effector from *Phytophthora infestans* prevents re‐localisation of two plant NAC transcription factors from the endoplasmic reticulum to the nucleus. PLoS Pathogens, 9, e1003670 10.1371/journal.ppat.1003670 24130484 PMC3795001

[eva12629-bib-0042] Menna, A. , Nguyen, D. , Guttman, D. S. , & Desveaux, D. (2015). Elevated temperature differentially influences effector‐triggered immunity outputs in Arabidopsis. Frontiers in Plant Science, 6, 995. 26617631 10.3389/fpls.2015.00995 PMC4637416

[eva12629-bib-0043] Mikkelsen, B. L. , Jørgensen, R. B. , & Lyngkjær, M. F. (2015). Complex interplay of future climate levels of CO_2_, ozone and temperature on susceptibility to fungal diseases in barley. Plant Pathology, 64, 319–327. 10.1111/ppa.12272

[eva12629-bib-0044] Nowicki, M. , Foolad, M. R. , Nowakowska, M. , & Kozik, E. U. (2012). Potato and tomato late blight caused by *Phytophthora infestans*: An overview of pathology and resistance breeding. Plant Disease, 96, 4–17. 10.1094/PDIS-05-11-0458 30731850

[eva12629-bib-0045] Nylander, J. A. A. (2008). MrModeltest v2.3. Program distributed by the author. Evolutionary Biology Centre, Uppsala University.

[eva12629-bib-0046] Oh, S. K. , Young, C. , Lee, M. , Oliva, R. , Bozkurt, T. O. , Cano, L. M. , … Morgan, W. (2009). In planta expression screens of *Phytophthora infestans* RXLR effectors reveal diverse phenotypes, including activation of the *Solanum bulbocastanum* disease resistance protein Rpi‐blb2. The Plant Cell, 21, 2928–2947. 10.1105/tpc.109.068247 19794118 PMC2768934

[eva12629-bib-0048] Ortega, E. , Bošković, R. I. , Sargent, D. J. , & Tobutt, K. R. (2006). Analysis of S‐RNase alleles of almond (*Prunus dulcis*): Characterization of new sequences, resolution of synonyms and evidence of intragenic recombination. Molecular Genetics and Genomics, 276, 413–426. 10.1007/s00438-006-0146-4 16924547

[eva12629-bib-0049] Phan, T. G. , Okitsu, S. , Maneekarn, N. , & Ushijima, H. (2007). Evidence of intragenic recombination in G1 rotavirus VP7 genes. Journal of Virology, 81, 10188–10194. 10.1128/JVI.00337-07 17609273 PMC2045391

[eva12629-bib-0050] Pilet, F. , Pellé, R. , Ellisseche, D. , & Andrivon, D. (2005). Efficacy of the R2 resistance gene as a component for the durable management of potato late blight in France. Plant Pathology, 54, 723–732.

[eva12629-bib-0051] Pond, S. L. K. , Frost, S. D. W. , Grossman, Z. , Gravenor, M. B. , Richman, D. D. , & Leigh Brown, A. J. (2006). Adaptation to different human populations by HIV‐1 revealed by codon‐based analyses. PLoS Computational Biology, 2, e62 10.1371/journal.pcbi.0020062 16789820 PMC1480537

[eva12629-bib-0052] Qin, C. F. , He, M. H. , Chen, F. P. , Zhu, W. , Yang, L. N. , Wu, E. J. , … Zhan, J. (2016). Comparative analyses of fungicide sensitivity and SSR marker variations indicate a low risk of developing azoxystrobin resistance in *Phytophthora infestans* . Scientific Reports, 6, 20483 10.1038/srep20483 26853908 PMC4745062

[eva12629-bib-0053] Raffaele, S. , & Kamoun, S. (2012). Genome evolution in filamentous plant pathogens: Why bigger can be better. Nature Reviews Microbiology, 10, 417–430. 10.1038/nrmicro2790 22565130

[eva12629-bib-0054] Raffaele, S. , Win, J. , Cano, L. M. , & Kamoun, S. (2010). Analyses of genome architecture and gene expression reveal novel candidate virulence factors in the secretome of *Phytophthora infestans* . BMC Genomics, 11, 637 10.1186/1471-2164-11-637 21080964 PMC3091767

[eva12629-bib-0055] Ronquist, F. , Teslenko, M. , Van Der Mark, P. , Ayres, D. L. , Darling, A. , Höhna, S. , … Huelsenbeck, J. P. (2012). MrBayes 3.2: Efficient Bayesian phylogenetic inference and model choice across a large model space. Systematic Biology, 61, 539–542. 10.1093/sysbio/sys029 22357727 PMC3329765

[eva12629-bib-0056] Sato, T. K. , Tremaine, M. , Parreiras, L. S. , Hebert, A. S. , Myers, K. S. , Higbee, A. J. , … Narasimhan, R. A. (2016). Directed evolution reveals unexpected epistatic interactions that alter metabolic regulation and enable anaerobic xylose use by *Saccharomyces cerevisiae* . PLoS Genetics, 12, e1006372 10.1371/journal.pgen.1006372 27741250 PMC5065143

[eva12629-bib-0057] Smyth, R. P. , Schlub, T. E. , Grimm, A. , Venturi, V. , Chopra, A. , Mallal, S. , … Mak, J. (2010). Reducing chimera formation during PCR amplification to ensure accurate genotyping. Gene, 469, 45–51. 10.1016/j.gene.2010.08.009 20833233

[eva12629-bib-0058] Städler, T. , & Delph, L. F. (2002). Ancient mitochondrial haplotypes and evidence for intragenic recombination in a gynodioecious plant. Proceedings of the National Academy of Sciences of the United States of America, 99, 11730–11735. 10.1073/pnas.182267799 12192087 PMC129337

[eva12629-bib-0059] Stefansson, T. S. , Willi, Y. , Croll, D. , & McDonald, B. A. (2014). An assay for quantitative virulence in *Rhynchosporium commune* reveals an association between effector genotype and virulence. Plant Pathology, 63, 405–414. 10.1111/ppa.12111

[eva12629-bib-0060] Stergiopoulos, I. , Cordovez, V. , Ökmen, B. , Beenen, H. G. , Kema, G. H. , & Wit, P. J. (2014). Positive selection and intragenic recombination contribute to high allelic diversity in effector genes of *Mycosphaerella fijiensis*, causal agent of the black leaf streak disease of banana. Molecular Plant Pathology, 15, 447–460. 10.1111/mpp.12104 24245940 PMC6638713

[eva12629-bib-0501] Tajima, F. (1989). Statistical method for testing the neutral mutation hypothesis by DNA polymorphism. Genetics, 123, 585–595. 2513255 10.1093/genetics/123.3.585 PMC1203831

[eva12629-bib-0061] Tamura, K. , Peterson, D. , Peterson, N. , Stecher, G. , Nei, M. , & Kumar, S. (2011). MEGA5: Molecular evolutionary genetics analysis using maximum likelihood, evolutionary distance, and maximum parsimony methods. Molecular Biology and Evolution, 28, 2731–2739. 10.1093/molbev/msr121 21546353 PMC3203626

[eva12629-bib-0062] Vetukuri, R. R. , Tian, Z. , Avrova, A. O. , Savenkov, E. I. , Dixelius, C. , & Whisson, S. C. (2011). Silencing of the PiAvr3a effector‐encoding gene from *Phytophthora infestans* by transcriptional fusion to a short interspersed element. Fungal Biology, 115, 1225–1233. 10.1016/j.funbio.2011.08.007 22115441

[eva12629-bib-0063] de Vries, S. , von Dahlen, J. K. , Uhlmann, C. , Schnake, A. , Kloesges, T. , & Rose, L. E. (2017). Signatures of selection and host‐adapted gene expression of the *Phytophthora infestans* RNA silencing suppressor PSR2. Molecular Plant Pathology, 18, 110–124. 10.1111/mpp.12465 27503598 PMC6638260

[eva12629-bib-0065] Wang, X. , Boevink, P. , McLellan, H. , Armstrong, M. , Bukharova, T. , Qin, Z. , & Birch, P. R. (2015). A host KH RNA‐binding protein is a susceptibility factor targeted by an RXLR effector to promote late blight disease. Molecular Plant, 8, 1385–1395. 10.1016/j.molp.2015.04.012 25936676 PMC4560694

[eva12629-bib-0066] Watt, W. B. (1972). Intragenic recombination as a source of population genetic variability. The American Naturalist, 106, 737–753. 10.1086/282809

[eva12629-bib-0067] Wu, E. J. , Yang, L. N. , Zhu, W. , Chen, X. M. , Shang, L. P. , & Zhan, J. (2016). Diverse mechanisms shape the evolution of virulence factors in the potato late blight pathogen *Phytophthora infestans* sampled from China. Scientific Reports, 6, 26182 10.1038/srep26182 27193142 PMC4872137

[eva12629-bib-0502] Yang, Z. (2007). PAML 4: Phylogenetic analysis by maximum likelihood. Molecular Biology and Evolution, 24, 1586–1591. 17483113 10.1093/molbev/msm088

[eva12629-bib-0068] Yang, L. , McLellan, H. , Naqvi, S. , He, Q. , Boevink, P. C. , Armstrong, M. , … Gilroy, E. M. (2016). Potato NPH3/RPT2‐like protein StNRL1, targeted by a *Phytophthora infestans* RXLR effector, is a susceptibility factor. Plant Physiology, 171, 645–657. 10.1104/pp.16.00178 26966171 PMC4854710

[eva12629-bib-0069] Yang, L. N. , Zhu, W. , Wu, E. , Yang, C. , Thrall, P. H. , Burdon, J. J. , … Zhan, J. (2016). Trade‐offs and evolution of thermal adaptation in the Irish potato famine pathogen *Phytophthora infestans* . Molecular Ecology, 25, 4047–4058. 10.1111/mec.13727 27288627

[eva12629-bib-0070] Zhan, J. , Linde, C. C. , Jürgens, T. , Merz, U. , Steinebrunner, F. , & McDonald, B. A. (2005). Variation for neutral markers is correlated with variation for quantitative traits in the plant pathogenic fungus *Mycosphaerella graminicola* . Molecular Ecology, 14, 2683–2693. 10.1111/j.1365-294X.2005.02638.x 16029470

[eva12629-bib-0071] Zhan, J. , Pettway, R. E. , & McDonald, B. A. (2003). The global genetic structure of the wheat pathogen *Mycosphaerella graminicola* is characterized by high nuclear diversity, low mitochondrial diversity, regular recombination, and gene flow. Fungal Genetics and Biology, 38, 286–297. 10.1016/S1087-1845(02)00538-8 12684018

[eva12629-bib-0072] Zhan, J. , Thrall, P. H. , & Burdon, J. J. (2014). Achieving sustainable plant disease management through evolutionary principles. Trends in Plant Science, 19, 570–575. 10.1016/j.tplants.2014.04.010 24853471

[eva12629-bib-0073] Zhan, J. , Thrall, P. H. , Papaïx, J. , Xie, L. H. , & Burdon, J. J. (2015). Playing on a pathogen's weakness: Using evolution to guide sustainable plant disease control strategies. Annual Review of Phytopathology, 53, 19–43. 10.1146/annurev-phyto-080614-120040 25938275

[eva12629-bib-0075] Zhu, S. , Li, Y. , Vossen, J. H. , Visser, R. G. F. , & Jacobsen, E. (2012). Functional stacking of three resistance genes against *Phytophthora infestans* in potato. Transgenic Research, 21, 89–99. 10.1007/s11248-011-9510-1 21479829 PMC3264857

[eva12629-bib-0076] Zhu, W. , Yang, L. N. , Wu, E. J. , Qin, C. F. , Shang, L. P. , Wang, Z. H. , & Zhan, J. (2015). Limited sexual reproduction and quick turnover in the population genetic structure of *Phytophthora infestans* in Fujian, China. Scientific Reports, 5, 10094 10.1038/srep10094 25970264 PMC4429539

